# Self-Approach Tendencies: Relations With Explicit and Implicit Self-Evaluations

**DOI:** 10.3389/fpsyg.2019.00309

**Published:** 2019-02-21

**Authors:** Lieke M. J. Swinkels, Hidde Gramser, Eni S. Becker, Mike Rinck

**Affiliations:** Behavioural Science Institute, Radboud University, Nijmegen, Netherlands

**Keywords:** approach-avoidance task, self-approach, self-portraits, self-evaluations, AAT, Rosenberg self-esteem scale, implicit association test

## Abstract

We used a newly developed Self-Approach-Avoidance Task (Self-AAT) to measure self-approach tendencies in female students. In this task, participants use a joystick to pull portraits of themselves and of others closer or to push them away. In the three studies, we found a significant self-approach tendency: participants were faster to pull mirror-imaged portraits of themselves closer than to push them away. This approach tendency was reduced for non-mirrored self-portraits, and absent for control pictures showing unknown males, unknown females, or empty backgrounds. Moreover, in two out of the three studies, the self-approach tendency was weakly related to explicit self-valuations measured with the Rosenberg Self-Esteem Scale (RSES), and in one out of two studies, it was related to implicit self-evaluations measured with the Implicit Association Task (IAT). Implications and potential applications of the findings are discussed.

## Introduction

Imagine a student named Sofie who lives on Sophia Road, and another one named Anne who lives on St. Anna Street. Even though student housing is always quite hard to come by, their choices for these specific streets may not be coincidental, and they may even be unaware of the reasons for their preferences (Nisbett and Wilson, 1977). Nuttin (1985) showed that these preferences may influence our choices, even if we are not consciously aware of them. In his Name Letter Task (NLT; Nuttin, 1985), most people prefer their own initials over the remaining letters of the alphabet. Thus, mere relatedness to the self can be a sufficient condition for enhancing the attractiveness of some alternatives. Sharing almost all name letters with the names of their streets, Sofie’s and Anne’s choices may not have been coincidental.

Our preference for some stimuli over others is not only reflected in our choices, but also in our approach-avoidance behavior. When people are presented with a positively valenced stimulus, approach movements are made faster than avoidance movements. In contrast, negatively valenced stimuli facilitate avoidance movements over approach movements, which were shown first by Solarz (1960). Since the attractiveness of self-associated stimuli is enhanced, a logical conclusion would be that they are more positively valenced than other-related stimuli, and that therefore they should facilitate approach behavior more than avoidance behavior, when compared to stimuli related to others. In the experiments reported here, we set out to test this assumption using stimuli that are highly associated with the self, namely, self-portraits.

In all three experiments, we employed self-portraits of the participants, in a task that taps into approach-avoidance responses to valenced stimuli: the Approach-Avoidance Task (AAT; Heuer et al., 2007; Rinck and Becker, 2007). The AAT is a joystick task in which participants have to respond to single pictures by pulling or pushing a joystick. When the joystick is pulled, the picture grows in size, when it is pushed, the picture shrinks. This creates a strong subjective impression of pulling the picture closer versus pushing it away. The task makes use of the compatibility between stimulus valence and movement direction: it is easier to pull pleasant objects closer and push unpleasant ones away than *vice versa* (Rinck and Becker, 2007). The AAT has been used to study approach-avoidance tendencies in many different domains, including spider phobia (Klein et al., 2011), social anxiety (Lange et al., 2008), addiction (Wiers et al., 2011; Eberl et al., 2013, 2014; Sharbanee et al., 2013), peer popularity (Lansu et al., 2012), post-traumatic stress disorder (Fleurkens et al., 2014), or skin picking (Schuck et al., 2012). Moreover, many studies employed pictures of faces as stimuli (Heuer et al., 2007; Roelofs et al., 2010; Voncken et al., 2012; Rinck et al., 2013; Vrijsen et al., 2013; Woud et al., 2013; Deckers et al., 2014). In studies of approach-avoidance tendencies in Social Anxiety Disorder, for instance, the participants encountered faces that looked neutral, angry, or happy, and they pulled them closer or pushed them away. However, in all of these studies, the participants never encountered pictures of themselves. For the present study, we designed a modified version of the AAT, the Self-Approach-Avoidance Task (Self-AAT). The Self-AAT uses portraits of the participants themselves, portraits of unknown others, and pictures of empty backgrounds to study approach-avoidance responses to the self, to others, and to neutral control stimuli. If the self-portrait is indeed, as hypothesized, a positively valenced stimulus for most humans, we should observe a self-approach tendency: compared to other facial and non-facial stimuli, participants should be faster to pull self-portraits closer than to push them away. In the Self-AAT, the pictures appear either in gray scale or in sepia. Participants are instructed to respond to the color of the pictures by pushing or pulling the joystick, thereby creating an avoidance or approach movement, respectively. RTs for full joystick movements are measured and used as the dependent variable. Participants are instructed to focus on the color of the pictures while ignoring their content.

Of course, we did not predict that all participants would show the postulated self-approach tendency to a similar degree. Instead, we expected the strength of the tendency to be related to the participants’ so-called self-esteem, defined as the overall affective evaluation of one’s own worth, value, or importance (Blascovich and Tomaka, 1991). In self-evaluations, a distinction is made between explicit versus implicit self-evaluations. Direct measures such as the Rosenberg Self-Esteem Scale (RSES; Rosenberg, 1965) measure explicit self-evaluations, that is, how people evaluate themselves at a deliberative, explicit level. In contrast, indirect measures assess implicit or spontaneous evaluations of the self (Greenwald and Banaji, 1995). Since both implicit self-evaluations and the self-approach tendency are hypothesized to draw on implicit processes related to the self, they should be related. In that case, the self-approach tendency should be larger for participants with more positive implicit self-evaluations. However, questionnaire measures of explicit self-evaluations tend to be more reliable than reaction time measures of implicit self-evaluations (Greenwald and Farnham, 2000); therefore, we may stand a better chance of finding a relation between self-approach and explicit self-evaluations, if there is one. In any case, it should be noted that the new Self-AAT was not intended to be another reaction time measure of implicit self-evaluations, designed to replace measures such as the so-called “Self-Esteem Implicit Association Test” (Glashouwer and De Jong, 2010). Instead, we aimed to test whether self-evaluations, both explicit and implicit, are related to the self-approach tendencies measured with the Self-AAT. Nevertheless, the Self-AAT might have a few advantages compared to the Implicit Association Task (IAT) and other measures of implicit self-evaluations: it uses pictorial rather than verbal stimuli, it does not require an opposing category (“Others”) in addition to the category of interest (“Self”), and it measures behavioral responses indicative of approach-avoidance tendencies.

In all three experiments, we tested the existence of the predicted self-approach tendency with the Self-AAT, and we related it to measures of explicit and implicit self-evaluations. More specifically, in Experiment 1, we demonstrated a significant self-approach tendency, and we related it to explicit self-evaluations. In Experiment 2, we replicated the self-approach tendency and related it to both explicit and implicit self-evaluations. In Experiment 3, we tested an explanation for the surprisingly large self-approach tendency found in Experiment 2, and we investigated the potential role of mere exposure contributing to the self-approach tendency.

## Experiment 1

The Self-AAT was designed to measure self-approach tendencies with several ideas in mind. First, Nuttin (1985) showed that we often prefer self-related items such as our initials. Using the Self-AAT, it is possible to assess whether we also show behavioral approach of ourselves, that is, of our own face. In the Self-AAT, the critical stimuli clearly refer to the participants’ self because they are portrait pictures of the participants themselves. By using these pictures, a high degree of self-relevance is created. Moreover, the responses to these self-portraits are compared to the responses to other portrait pictures and to neutral control pictures. Second, we aimed to find out whether the self-approach tendency measured with the Self-AAT correlates with measures of explicit and implicit self-evaluations. If the strength of the self-approach tendency is indeed related to how positively the self is evaluated, either explicitly or implicitly, then participants with more positive self-evaluations should show a larger self-approach tendency.

The aim of the first experiment was to investigate whether the Self-AAT can be used to assess self-approach tendencies, and whether these tendencies are related to self-evaluations. For the latter goal, we aimed to compare the Self-AAT to established measures of explicit self-evaluations (the RSES) and implicit self-evaluations (the NLT). Unfortunately, due to a programming error in the NLT procedure, the participants’ initials were not saved. Therefore, it was impossible to determine beyond doubt how they evaluated their initials compared to the remaining letters of the alphabet. As a result, we do not report on the NLT here.

### Methods

We fully disclose details of data collection, data exclusions, all manipulations and all measures in this and the following two studies. The studies were approved by the Ethics Committee of the Faculty of Social Sciences (ECSW) of Radboud University.

#### Participants

In this experiment, 109 female students of Radboud University Nijmegen participated for course credit (age: *M* = 20.5, *SD* = 1.5, range = 18–26 years). Testing the main hypothesis of this study involved a 2 × 4 within-subjects interaction. In the absence of previous studies from which effect sizes could be estimated, we powered the study for a medium-sized effect of this interaction (*f* = 0.25), yielding excellent power of 1 − β = 0.99 at *p* = 0.05 (computed with G*Power 3.1; Faul et al., 2007). Similarly, the power to detect a medium-sized correlation of the hypothesized self-approach tendency with explicit or implicit self-evaluations (*r* = 0.30) at *p* = 0.05 was also excellent (1 − β = 0.94).

#### Rosenberg Self-Esteem Scale

As in many previous studies, the RSES (Rosenberg, 1965; Franck et al., 2008) was used to measure explicit self-evaluations. The RSES consists of 10 statements designed to assess various aspects of self-evaluations.

#### Self-Approach-Avoidance Task

Previous studies have indicated that the AAT is a valid way to measure approach-avoidance tendencies in response to facial stimuli (Heuer et al., 2007). As in most of the earlier studies, the present experiment employed an indirect version of the AAT: participants had to respond to the color of the pictures instead of their content. Participants were instructed to pull the joystick in response to all gray-scale pictures and to push the joystick in response to all sepia-colored pictures. By moving the joystick, two cues of approach and avoidance were combined: the physical arm movements, and a concurrent increase or decrease of the picture size. A medium-sized picture was shown first which increased or decreased dynamically in size, depending on the joystick movements. Pictures only disappeared after a full joystick movement in the correct direction.

The stimulus pictures for the Self-AAT consisted of three faces of unknown males, three faces of unknown females (all in the same age range as the participants), one picture of an empty background (the slightly textured white blanket in front of which all other pictures were taken), and three pictures of the participant’s face herself. The latter pictures were mirrored around the vertical axis, such that the participants would see themselves as they usually see themselves in a mirror. A gray-scale version and a sepia version were created of each picture. All pictures were taken with the same camera, a Canon Digital Ixus 60, at the highest resolution, without a flash. Of each picture, seven different sizes were created for the zooming effect. Pictures were first set to a height of 1,440 pixels to match the resolution of the screen on which the task was presented. This maximum-size picture was then decreased in size to 70% of its original size and saved, in order to create the second largest picture. This procedure was repeated until seven picture sizes were created.

The Self-AAT started with a practice block of 12 empty control pictures, during which the experimenter remained with the participant to provide guidance. When the participant had no further questions, the experimenter left and the experimental block was started. The block consisted of two more practice trials and 96 experimental trials, and it lasted for approx. 5 min. The experimental trials showed self-portraits, unknown females, unknown males, and empty background controls. Each of these four picture types was pulled closer 12 times and pushed away 12 times. The trials were presented in a quasi-random order with the restriction that no more than two trials of the same type followed each other. The Self-AAT was completed on a Pentium III computer running Windows XP with a 15″ screen. A Logitech Attack 3 joystick, firmly attached to the table in front of the computer screen, was used to respond to the pictures.

#### Procedure

Upon arrival at the lab, the participant was informed that the experiment required that pictures of her were taken, but that for privacy reasons, the pictures would be deleted after the experiment. After receiving these instructions, she was asked to read and sign an informed consent form which described the task as investigating “reactions to pictures of people.” Next, the pictures for the Self-AAT were taken. The participant was asked to stand in front of a wall with the background blanket on it, and to look friendly, but without showing teeth. The models for the pictures of unknown males and females had received identical instructions. Three pictures were taken of each participant. Then, the participant completed the RSES and several other questionnaires unrelated to this study. The latter were only included to give the experimenter enough time to prepare the participant’s pictures for the Self-AAT. When the participant had finished the questionnaires, the Self-AAT was started, followed by the NLT. Finally, the participant was extensively debriefed and thanked for her participation. In total, the experiment lasted for approximately 30 min.

### Results

#### Rosenberg Self-Esteem Scale

On average, participants scored high on explicit self-evaluations, as measured with the RSES (*M* = 32.5, *SD* = 4.9, range = 21–40).

#### Self-Approach-Avoidance Task Reaction Times

To prepare the Self-AAT reaction time data for analysis, the RTs of the correct final movements were used. As in earlier AAT studies, we first excluded the 1% fastest and the 1% slowest reactions from these RTs, in order to exclude potential extreme outlier RTs on both ends of the RT distribution. From the remaining trials, median RTs were computed for each participant, separately for each of the eight combinations of movement and picture type. As in previous AAT studies, medians were computed rather than means because they are more robust against outliers. Then, boxplots were used to identify participants with extreme values of these median RTs. This revealed one participant who had outlying scores in three out of the eight combinations (significantly longer RTs than the rest of the sample). Therefore, this participant was excluded, and the final sample consisted of 108 participants. The resulting means per condition are shown in Table 1.

**Table 1:** Mean reaction times and standard deviations in ms per picture type and motion in Experiments 1 and 2.

Picture type	Motion	Mean	*SD*
		Exp. 1	Exp. 2	Exp. 1	Exp. 2
Empty control	Pull	646	744	105	127
Empty control	Push	620	683	99	114
Unknown male	Pull	693	780	139	171
Unknown male	Push	661	718	112	129
Unknown female	Pull	681	759	139	169
Unknown female	Push	656	711	127	126
Mirrored self	Pull	639	740	119	158
Mirrored self	Push	658	1,077	131	309

The median RTs were analyzed using a Picture Type (self, unknown-male, unknown-female, empty-control) × Movement Direction (pull, push) repeated-measures ANOVA. The ANOVA yielded significant main effects of picture type, *F*(3,105) = 21.59, *p* < 0.001, $\eta_{p}^{2}=0.38,$ and of movement direction, *F*(1,107) = 11.64, *p* = 0.001, $\eta_{p}^{2}=0.10,$ indicating that the pushing movement was generally made faster than the pulling movement, and that participants generally responded fastest to the empty-control stimuli, followed by self, unknown-female, and unknown-male. Most importantly, a large and significant interaction effect of picture type and movement direction was found, *F*(3,105) = 6.84, *p* < 0.001, $\eta_{p}^{2}=0.16.$ Participants were faster to push than to pull faces of males, *t*(107) = 3.37, *p* = 0.001, *d* = 0.25, faces of females, *t*(107) = 2.91, *p* = 0.004, *d* = 0.19, and empty control pictures, *t*(107) = 3.98, *p* < 0.001, *d* = 0.26. In contrast, they pulled pictures of themselves closer more quickly than they pushed them away, *t*(107) = 2.12, *p* = 0.037, *d* = 0.15. Therefore, compared to all other stimuli, participants showed an approach tendency towards their own faces only.

#### Correlations of Self-Approach-Avoidance Task Scores With Rosenberg Self-Esteem Scale

To correlate the Self-AAT results with the RSES, Self-AAT scores were computed first. To this end, each participant’s median RT for pulling self-portraits was subtracted from her median RT for pushing them. Positive values suggest self-approach, and negative values suggest self-avoidance. The same RT difference was also computed for unknown males, unknown females, and empty control pictures. Then, three relative Self-AAT scores were computed by subtracting the RT difference of empty controls from each of the three other RT differences. For instance, the Self-AAT score for self-portraits was computed according to the formula [(self-push − self-pull) − (control-push − control-pull)], and positive values indicate that the self-approach tendency exceeds that for empty control stimuli. This procedure yielded three different AAT scores, allowing us to determine self-approach tendencies independently of other-male-approach tendencies and other-female-approach tendencies. Correlating these AAT scores with the RSES scores resulted in a significant positive correlation between the Self-Control score and the RSES, *r* = 0.21, *p* = 0.03, indicating that a stronger self-approach tendency was accompanied by more positive explicit self-evaluations. No correlation was found between the RSES and the Male-Control scores, *r* = −.04, *n.s.*, or the Female-Control scores, *r* = −.16, *n.s.*, indicating that a stronger other-avoidance tendency was not related to self-evaluations.

### Discussion

The aims of the first experiment were to establish the existence of the predicted self-approach tendency independently of possible other-avoidance tendencies and to investigate whether the self-approach tendency was related to explicit self-evaluations.

To be indicative of a significant self-approach tendency that exceeds the corresponding tendencies for other stimuli, the RT pattern for self-portraits should differ from the patterns of other stimuli. This was the case: the participants of Experiment 1 were faster to approach than to avoid self-portraits, whereas the opposite was observed for faces of unknown males and females, as well as empty control pictures. The latter pictures are particularly important because they can be used to establish a baseline for approach-avoidance tendencies: in order to be meaningful, any observed approach tendency must be significantly larger than the one found for the meaningless, empty control pictures. This was indeed the case in Experiment 1. Then, different Self-AAT scores were computed and correlated with a frequently used measure of explicit self-evaluations, the RSES. A correlation was indeed observed between the RSES and the self-approach scores, but not between the RSES and the unknown-male or unknown-female scores. This suggests that it is indeed the reaction to self-portraits which is related to explicit self-evaluations, not the reaction to portraits of others. The observed correlation was rather weak, however, which is compatible with our assumption that explicit self-evaluations and the self-approach tendency are related, but different, constructs.

## Experiment 2

In Experiment 2, we aimed to replicate the self-approach tendency found in Experiment 1. Furthermore, we also wanted to study the relation between the self-approach tendency and implicit self-evaluations in addition to its relation with explicit self-evaluations, measured with the RSES. Unlike Experiment 1, however, we chose to employ the so-called “Self-Esteem Implicit Association Task” [SE-IAT, henceforth Implicit Association Task (IAT)] as a measure of implicit self-evaluations instead of the NLT because this IAT seems to be more reliable (Krause et al., 2011), and because it has been used in many previous studies (De Raedt et al., 2006; Franck et al., 2008; Glashouwer and De Jong, 2010). Since both the IAT and the Self-AAT are indirect measures, we were interested to investigate whether the Self-AAT would correlate more strongly with the IAT than with the RSES, even though questionnaires are usually more reliable than RT measures.

### Methods

Experiment 2 was very similar to Experiment 1, therefore, only the differences will be described.

#### Participants

Eighty-six female students of Radboud University Nijmegen participated in this experiment for course credit (age: *M* = 19.5, *SD* = 1.5, range = 17–28 years). Despite the large interaction effect found in Experiment 1, we powered this study for a medium-sized interaction effect as well (*f* = 0.25), to protect ourselves against potential random inflation of the effect in Experiment 1. The sample size yielded an excellent power of 1 − β = 0.98 to detect a medium-sized 2 × 4 interaction effect at *p* = 0.05. The power to detect a medium-sized correlation of the observed self-approach tendency with explicit or implicit self-evaluations (*r* = 0.30) at *p* = 0.05 was also good, with 1 − β = 0.88 (Faul et al., 2007).

#### Self-Approach-Avoidance Task

The task was identical to the one used in Experiment 1. A minor change included the use of a different camera to take the participant pictures. Here, a HP Photosmart R607 camera was used to take the participants’ pictures. The other pictures remained unchanged.

#### Implicit Association Task

The IAT is a computerized reaction time task designed to measure the relative strengths of the associations of two contrasted target concepts (here: Me versus Others) with two valenced attribute concepts (here: Positive versus Negative). Although there is considerable debate about what IATs actually measure (e.g., Remue et al., 2014), this IAT variant was designed to measure implicit self-evaluations, and it is frequently used as such. The version used here was very similar to the task employed by Glashouwer and de Jong (2010). Single words from the four categories appeared in a pseudo-random order on the screen, and participants were instructed to sort target words into the “Me” versus “Others” categories, and attribute words into the “Positive” versus “Negative” categories. In both cases, they used either the left response key (“E”) or the right response key (“I”). To avoid confusion, the category and attribute labels were visible in the upper left and right corner during the entire experiment. Each category consisted of five Dutch words. They were equivalent to the English words I, myself, self, my, and own (Me); other, you, they, them, and themselves (Others); useful, successful, important, valuable, and self-assured (Positive); and unimportant, useless, weak, failure, and worthless (Negative).

The IAT contained two critical test blocks of 40 trials each, in which targets and attributes were categorized together. In the first test block, “Me” words and “Positive” words shared the same response key, while “Others” words and “Negative” words shared the other response key. In the second test block, the allocation of targets to response keys was reversed, such that “Other” words and “Positive” words now shared the same response key, and “Me” words and “Negative” words shared the other response key. The assumption behind this task is that sorting should be easier and faster when strongly associated targets and attributes share the same response key. Thus, participants who associate positive rather than negative attributes with themselves should respond more quickly in the “Me-Positive” block than in the “Me-Negative” block.

Each trial started with a fixation cross that remained on the screen for 200 ms. Then, the word was presented, and participants had 3,000 ms to respond. When participants were too slow or gave an incorrect response, they received the message “Wrong or too slow”, after which the trial automatically ended. After 500 ms, a new trial started automatically. To reduce error variance, the order of the two test blocks and the order of trials within each block were held constant across participants.

#### Procedure

While the experimenter prepared the pictures for the Self-AAT, the participant completed the RSES and the IAT. The experiment took approximately 30 min.

### Results

#### Rosenberg Self-Esteem Scale

On average, participants scored high on explicit self-evaluations, as measured with the RSES (*M* = 32.6, *SD* = 4.4, range = 17–40).

#### Self-Approach-Avoidance Task

The Self-AAT data were prepared for analysis in the same manner as in Experiment 1. No participant had to be excluded. The same Picture Type × Movement Direction ANOVA was conducted on the data, and the results closely resembled those of the first experiment. Again, we found significant main effects of picture type, *F*(3,83) = 54.90, *p* < 0.001, $\eta_{p}^{2}=0.67,$ and of movement direction, *F*(1,85) = 17.61, *p* = 0.001, $\eta_{p}^{2}=0.17,$ indicating that on average, the participants pulled more quickly than they pushed, and they generally responded fastest to the empty control stimuli, followed by unknown-females, unknown-males, and self-portraits. Most importantly, the significant interaction of picture type and movement direction was replicated, *F*(3,83) = 59.68, *p* < 0.001, and it was a very large effect, $\eta_{p}^{2}=0.68.$ Again, participants were faster to push than to pull faces of males, *t*(85) = 3.77, *p* < 0.001, *d* = 0.40, faces of females, *t*(85) = 3.22, *p* = 0.002, *d* = 0.31, and empty control pictures, *t*(85) = 5.53, *p* < 0.001, *d* = 0.50, whereas they pulled pictures of themselves closer more quickly than they pushed them away, *t*(85) = 11.87, *p* < 0.001, *d* = 1.25. Therefore, the self-approach tendency observed in Experiment 1 was replicated here. The only noticeable difference between the two experiments was observed for the speed of pushing self-portraits away: unlike Experiment 1, participants of Experiment 2 were unusually slow in this condition, yielding a mean RT that was approx. 300 ms slower than in all other conditions, thereby inflating the observed self-approach tendency. However, additional tests showed that this slowdown was not caused by outlier RTs or outlier participants.

#### Implicit Association Task

To prepare the IAT data for analyses, we computed the so-called D4 score, which was recommended by Greenwald et al. (2003) and has also been used in previous analyses of IAT data (e.g., Glashouwer and de Jong, 2010). Simply speaking, the D4 score standardizes each participant’s mean RT difference between the “Me-Positive” test block and the “Me-Negative” test block by dividing the RT difference by the standard deviation of the participant’s RTs, after correcting for errors and outlier RTs. The mean D4 score observed here was significantly positive, suggesting positive implicit self-evaluations [*M* = 0.70, *SD* = 0.42, *t*(85) = 15.58, *p* < 0.001].

#### Correlations of Self-Approach-Avoidance Task With Rosenberg Self-Esteem Scale and IAT

A significant correlation was found between RSES scores and IAT scores, *r* = 0.26, *p* < 0.02. Next, we related the self-approach tendencies observed in the Self-AAT to the RSES scores. A marginally significant correlation with the RSES was found when the self-tendency was computed by comparing self-portraits to empty control pictures, *r* = 0.189, *p* = 0.083. When comparing self-portraits to unknown females, basically the same correlation with the RSES was observed, *r* = 0.187, *p* = 0.086. When compared to unknown males, the correlation with the RSES was a little higher and significant, *r* = 0.22, *p* < 0.05. Taken together, these results suggest that the self-approach tendency was only weakly associated with more positive explicit self-evaluations. With regard to implicit self-evaluations, we found significant positive correlations of the AAT scores with the IAT when the self-tendency was computed by comparing self-portraits to unknown males, *r* = 0.30, *p* = 0.005, or to unknown females, *r* = 0.26, *p* = 0.015, and a marginally significant correlation when compared to empty control pictures, *r* = 0.21, *p* < 0.06. This pattern suggests that a stronger self-approach tendency was weakly associated with more positive implicit self-evaluations, as measured with the IAT.

### Discussion

The aims of Experiment 2 were to replicate the findings of Experiment 1 and to extend these findings to implicit self-evaluations. First, we did indeed replicate the self-approach tendency observed before: unlike all other stimuli, participants approached their own mirror-imaged faces more quickly than they avoided them. The correlation with the RSES observed here (*r* = 0.189) was almost identical to the one observed in Experiment 1 (*r* = 0.21). However, due to the slightly smaller sample size, this small-to-medium correlation was only marginally significant in Experiment 2. Moreover, the RSES also correlated with the IAT. Relating the different Self-AAT scores to IAT effects resulted in significant and marginally significant correlations. These findings suggest that the self-approach tendency can indeed be seen as weakly related to both implicit and explicit self-evaluations. The small correlation between the IAT and the RSES is also worth noting. One might wonder, however, whether this should be considered problematic. Several authors have stated that explicit and implicit self-evaluations may be two different constructs, which implies that they do not have to correlate substantially with each other (Karpinski, 2004; Franck et al., 2008; Oakes et al., 2008). The third experiment was designed to shed more light on this.

Another reason to conduct the third experiment was the surprisingly large difference between RTs for pulling and pushing the self-portraits. As Table 1 shows, this large difference is due to the unusually long RTs for pushing self-portraits away. The latter might be explained by the fact that a new camera was used to take participant pictures and that a new computer program was used to create the gray-scale and sepia versions of these pictures, whereas all other pictures were taken over from Experiment 1. Gray-scale pictures will look approximately the same regardless of the camera and program used, whereas sepia pictures might turn out differently because there is no generally binding definition of “sepia”. As a result, for the self-portraits it might have taken longer to decide that the picture was sepia, thereby resulting in larger RTs for pushing them away, and therefore a seemingly larger self-approach tendency. It is important to note, however, that such an artificial inflation of the self-approach tendency does not render it invalid: the tendency still correlated with the IAT, supporting its validity. Nevertheless, the third experiment addressed this problem directly.

## Experiment 3

In the third experiment, we attempted to overcome the possible flaw discussed above by using the same camera and computer program for all pictures. Moreover, a specific aspect of the task, namely face orientation, was investigated more closely. Since the development of mirrors, humans have been able to look at their own faces frequently, and they probably do this much more often than looking at portrait photographs of themselves. This has led us to perceive our normally oriented face as less familiar and possibly harder to recognize than our mirror-oriented face, an effect that is reversed for the faces of other people (Rhodes, 1986). Therefore, Dieguez et al. (2011) highlight the importance of taking face orientation into account when using faces as stimulus materials. And indeed, Mita et al. (1977) found that the participants of their study liked their mirror-image better than their non-mirrored portrait, while their friends and lovers showed the opposite preference. Mita et al. (1977) refer to the mere exposure effect (Zajonc, 1980) to explain this phenomenon: all the tested individuals preferred what they had been exposed to more often. This was the normally oriented face for friends and lovers, and the mirror-imaged face for the participants themselves. Given this preference for the mirrored self-portrait, we hypothesized that these images should also elicit a stronger self-approach tendency than the normally oriented portraits.

In Experiments 1 and 2, only mirror-imaged self-portraits were used, while the normally oriented portraits have not been studied yet. This has implications for our understanding of the factors underlying the observed self-approach tendency. Both portrait versions differ from other stimuli by depicting the self and by being more familiar than almost all other possible stimuli. Thus, both versions are high in what might be called *conceptual familiarity*, and they both depict an individual the participant knows very well. The two versions differ, however, in what might be called *perceptual familiarity*: in the past, the participant has seen her mirror-imaged portrait much more often than her normal portrait, which may then cause higher familiarity and liking, and an increased self-approach tendency.^1^ Experiment 3 was designed to determine the importance of conceptual and perceptual familiarity for the self-approach tendency. If the tendency is determined solely by conceptual familiarity, both original and mirror-imaged self-portraits should evoke the same self-approach tendency. If, on the other hand, perceptual familiarity is critical, only the mirror-imaged portrait should evoke a self-approach tendency. Finally, if both types of familiarity are important, we should observe a significant tendency for normally oriented self-portraits, and an even stronger tendency for mirror-imaged ones. To test these predictions, we presented both versions of the self-portraits to the participants. Furthermore, the correlations with the RSES and the IAT were investigated once more.

### Methods

Again, the experiment was very similar to the previous ones; therefore, only the differences will be described.

#### Participants

Ninety-five female students of Radboud University Nijmegen participated in this experiment (age: *M* = 20.1, *SD* = 1.9, range = 18–27 years). Similar to the previous experiments, this sample size yields an excellent power of 1 − β = 0.99 to detect a medium-sized 2 × 5 interaction effect at *p* = 0.05 and a very good power of 1 − β = 0.91 to detect a medium-sized correlation of the observed self-approach tendency with explicit or implicit self-evaluations (*r* = 0.30) at *p* = 0.05 (Faul et al., 2007).

#### Rosenberg Self-Esteem Scale and Implicit Association Task

The RSES and the IAT were identical to those of Experiment 2.

#### Self-Approach-Avoidance Task

For the present Self-AAT, a new computer and camera were used to create the participant pictures and new control pictures. The task was displayed on a Windows XP laptop with a 17″ screen. To ensure that all pictures had the same color scheme, the same camera, a Samsung S860, was used to create all stimuli. Furthermore, all confederates and participants were asked to show a neutral expression. As before, the pictures showed empty backgrounds, unknown males, unknown females, and the participant herself. Of the latter self-portraits, both the original and the mirror-imaged version were presented.

#### Procedure

Upon arrival at the lab, participants were informed that the experiment consisted of a faces part and a memory part (the unrelated N-back task described below). After completing the RSES, the IAT, and the Self-AAT for this study, the participants also completed a mood thermometer and a modified version of the N-back task in which negative feedback was implemented (Jaeggi et al., 2010). These were part of a different experiment and not related to the present experiment; therefore, they will not be discussed further. Finally, participants were extensively debriefed and thanked for their participation. The experiment took approximately 45 min.

### Results

Two participants were excluded from further analyses because boxplots showed that they produced significant outlier data on at least three variables of the Self-AAT, RSES, and IAT. Therefore, the final sample consisted of 93 participants.

#### Rosenberg Self-Esteem Scale

On average, participants scored high on explicit self-evaluations, as measured with the RSES (*M* = 30.3, *SD* = 4.4, range = 18–39).

#### Self-Approach-Avoidance Task

The Self-AAT data were prepared for analysis in the same manner as in the previous experiments. The median RTs were analyzed using a Picture Type (mirrored self, original self, male, female, and empty control) × Movement Direction (push, pull) repeated-measures ANOVA. This yielded a significant main effect for picture type, *F*(4,89) = 15.51, *p* < 0.001, $\eta_{p}^{2}=0.41,$ indicating that participants generally responded fastest to the empty control pictures, followed by original self, males, females, and mirrored self. No significant main effect was found for movement direction, *F*(1,92) = 1.98, *p* = 0.163. Most importantly, a significant interaction of picture type and movement direction was found, *F*(4,89) = 4.70, *p* = 0.002, $\eta_{p}^{2}=0.17.$ In Table 2, the mean reaction times for each picture type and movement direction are shown. Of particular interest is the approach-avoidance pattern that was found for the self-portraits: these were the only pictures for which we observed faster pulling than pushing. Two additional 4 × 2 ANOVAs indicated that the self-approach tendency for both the mirrored and the original self-portraits differed significantly from the pattern observed for male, female, and empty control pictures, yielding significant 2 × 4 interactions [mirrored: *F*(3,90) = 5.74, *p* = 0.001, $\eta_{p}^{2}=0.16;$ original: *F*(3,90) = 4.21, *p* = 0.008, $\eta_{p}^{2}=0.12].$ Moreover, the self-approach tendency for mirrored self-portraits was not significantly larger than the self-approach tendency for original self-portraits, *F*(1,92) = 1.66, *p* = 0.20, $\eta_{p}^{2}=0.02.$ Additional *t* tests, however, revealed that the difference between pulling and pushing (i.e., the self-approach tendency) was medium-sized and significant for the mirrored self-portraits, *t*(92) = 2.70, *p* = 0.008, *d* = 0.24, whereas it was only small and not significant for the original self-portraits, *t*(92) = 1.40, *p* = 0.165, *d* = 0.12.

**Table 2:** Mean reaction times and standard deviations in ms per picture type and motion in Experiment 3.

Picture type	Motion	Mean	*SD*
Empty control	Pull	580	71
Empty control	Push	571	75
Unknown male	Pull	606	107
Unknown male	Push	575	86
Unknown female	Pull	606	117
Unknown female	Push	598	72
Mirrored self	Pull	600	84
Mirrored self	Push	621	98
Original self	Pull	576	76
Original self	Push	585	80

#### Implicit Association Task

The data of the IAT were analyzed in the same way as in Experiment 2. Again, the mean D4 score was significantly positive, suggesting positive implicit self-evaluations [*M* = 0.77, *SD* = 0.48, *t*(92) = 15.31, *p* < 0.001].

#### Correlations of Self-Approach-Avoidance Task With Rosenberg Self-Esteem Scale and Implicit Association Task

Unlike Exp. 2, no significant correlation was found between the RSES scores and the D4 scores of the IAT, *r* = 0.04, *p* = 0.74. Next, we compared the Self-AAT to the IAT and the RSES, using the same procedure as above. None of the comparisons of mirrored-self versus control stimuli (male, female, empty-control) resulted in a significant correlation with the IAT (Self-Control: *r* = 0.03, *p* = 0.75; Self-Female: *r* = 0.08, *p* = 0.44; Self-Male: *r* = 0.01, *p* = 0.93). The same was true for the original self-portraits: Self-Control: *r* = 0.04, *p* = 0.73; Self-Female: *r* = 0.08, *p* = 0.42; Self-Male: *r* = 0.08, *p* = 0.42. Similarly, none of these six comparisons showed a significant correlation with the RSES either (all *r* < 0.18, *p* > 0.10). In sum, Exp. 3 did not reveal any significant correlation between self-approach tendencies, explicit self-evaluations, and implicit self-evaluations.

### Discussion

Experiment 3 was designed to gain a better understanding of the Self-AAT in two ways: first, a specific aspect of the task, namely face orientation, was studied in more detail, and second, we tried to replicate the previously found correlations between the Self-AAT and the RSES and IAT. Furthermore, we tested whether the surprisingly large self-approach tendency observed in Experiment 2 could be explained by the use of different cameras and computer programs for self-portraits versus control pictures.

For the mirrored self-portraits, we replicated the self-approach tendency observed in the first two experiments: participants pulled them closer more quickly than they pushed them away. Moreover, now that all images were taken with the same camera and processed in the same way, making all sepia tones look similar, the self-approach tendency was no longer inflated. Instead, the current size of the self-approach tendency for mirrored self-portraits resembled that of Experiment 1.

Interestingly, the self-approach tendency was not statistically significant for the original self-portraits. However, it was significantly different from the avoidance tendencies observed for male, female, and empty control pictures. Moreover, the self-approach tendency for original self-portraits was not significantly smaller than the one for the mirrored self-portraits. These findings suggest that both conceptual and perceptual familiarity are involved in evoking a reliable self-approach tendency: the tendency is largest when the picture shows my face as I am used to seeing it frequently.

Finally, none of the previously found correlations between RSES, IAT, and Self-AAT could be replicated, making it hard to decide how the Self-AAT is related to implicit and explicit self-evaluations. We will address this point in the General Discussion section below.

## General Discussion

Taken together, the three experiments presented here established the existence of the predicted self-approach tendency in the new task, the Self-AAT. In all three experiments, participants were faster to approach than to avoid mirror-imaged pictures of themselves. The opposite pattern occurred for unknown males, unknown females, and empty control stimuli. Thus, we seem to have found a specific relation between self-portraits and approach. For most of us, the self is a positive stimulus, which makes approach easier and avoidance harder (Banaji and Prentice, 1994).

In addition, Experiment 3 provided support for the hypothesis that the self-approach tendency is driven by both conceptual familiarity (“it is me”) and perceptual familiarity (“it looks like me”). Both types of self-portraits yielded approach tendencies that differed from the avoidance tendencies observed for the control stimuli. However, only mirrored self-portraits yielded a statistically significant self-approach tendency, which highlights the importance of perceptual familiarity and of mirroring participant pictures (Dieguez et al., 2011). It should be noted, however, that this result does not fully qualify for the mere exposure effect cited by Mita et al. (1977): in order for an exposure effect to be a mere one, participants should be unable to report what was presented to them. However, our participants were certainly aware of what they saw every time they looked into a mirror. Nevertheless, our results speak to the importance of perceptual familiarity caused by frequent prior exposure.

The fact that a strong self-approach tendency was only found for mirrored self-portraits might suggest that these are also more positive or pleasant than non-mirrored self-portraits. This is consistent with the results reported by Mita et al. (1977) and should be assessed in future studies. Moreover, it should be tested if the association between mirrored self-images and approach tendencies originates from repeated previous behavior: we often approach our mirror image to take a closer look at ourselves in the mirror, possibly making this the dominant reaction to mirror images of ourselves. This everyday movement towards a mirror is obviously different from pulling the mirror image closer by hand, but both movements imply approach.

The results regarding the correlations of the Self-AAT with the RSES and the IAT were fairly inconclusive: a significant correlation of the Self-AAT and the RSES was found in Exp. 1, a marginally significant one in Exp. 2, and none in Exp. 3. A significant correlation between the Self-AAT and the IAT was obtained in Exp. 2, but not in Exp. 3. As discussed above, weak correlations between the RSES questionnaire and the two response-time measures Self-AAT and IAT are not necessarily problematic because they are meant to address different aspects of self-evaluations: explicit versus implicit (Karpinski, 2004; Franck et al., 2008; Oakes et al., 2008). In contrast, the lack of a correlation between the Self-AAT and the IAT in Experiment 3 is more problematic if one assumes that the self-approach tendency in the Self-AAT results from the positive self-attitude that underlies implicit self-evaluations. However, it might be generally difficult to find correlations between indirect measures. For instance, Krause et al. (2011) demonstrated that the IAT and the NLT did not correlate with each other either, even though both are supposed to measure implicit self-evaluations. A straightforward explanation of this may be insufficient reliability, since both Self-AAT scores and IAT scores are based on differences of reaction times. However, an alternative and maybe more likely explanation could be that self-evaluations and self-approach are different and only loosely related constructs, referring to associations versus behavioral tendencies, respectively. More reliable measures and more research into this topic are needed to find the most likely explanation. However, the fact that an approach tendency exists for our own mirrored portrait is interesting in its own right, even if the Self-AAT would turn out to be unrelated to self-evaluations.

Could mere familiarity explain the self-approach tendency observed in the three experiments reported here? That is, would any familiar stimulus evoke the approach tendency observed for mirror-imaged self-portraits? The current experiments were not designed to test this hypothesis, but we expect the answer to be No. Familiarity undoubtedly contributes to preferences, as evidenced by the mere exposure effect investigated intensively by Zajonc and others (e.g., Zajonc, 1980). Thus, one might expect familiar stimuli to evoke an approach tendency. However, approach tendencies seem to require positive valence, too, because there is evidence of avoidance despite familiarity, for instance, avoidance of feared objects by phobics (Rinck and Becker, 2007), as well as evidence for valence-related differences in approach-avoidance tendencies despite similar levels of familiarity (e.g., Lansu et al., 2012). Thus, it seems that familiarity will cause an approach tendency only if the familiar stimuli are also positive, pleasant stimuli. Nevertheless, it would be informative to find out whether strongly positive pictures elicit an approach tendency that is comparable to the self-approach tendency observed here. Additionally, it would be informative to use familiar others like family or friends as control stimuli instead of unknown others.

Some limitations should also be noted. Most importantly, the samples in all three experiments consisted of highly educated females, a group for which it is likely that on average, they have positive self-evaluations, both explicit and implicit. This limitation to female student samples causes two problems: first, the variance in self-approach tendency, explicit self-evaluations, and implicit self-evaluations might be reduced, thereby also reducing the likelihood of finding correlations between these measures. Second, we cannot be sure whether self-approach tendencies of similar size would be observed in other populations, for instance, in males. Males are less likely than females to have body image problems, therefore, they may show an even stronger self-approach tendency, particularly when whole-body pictures are used. In contrast, some individuals, for instance, depressed patients, might not show self-approach at all, or maybe even self-avoidance. Future studies need to address this knowledge gap by applying the Self-AAT in more heterogeneous groups with more variance in mood, self-evaluations, age, education, and gender.

Other limitations involve the fixed order of the tasks and the valence of the facial expressions used here. Because of the fixed order, we cannot determine or exclude effects of the self-evaluations measures on the Self-AAT. The valence may be important because we did not measure how friendly or positive the participants’ faces looked, compared to the confederates’ faces, and we did not collect pre-experimental ratings of the control picture’s valence.

Another limitation arises from the fact that we did not systematically assess the participants’ task awareness. Therefore, we do not know how many of them could correctly guess the aim of the studies. During debriefing, however, no participant expressed awareness of the main hypothesis (stronger self- than other-approach) or awareness of the expected correlation with self-evaluations. Furthermore, a practical limitation of the Self-AAT is that for each participant, an individual version has to be created with his or her portraits. On the other hand, these self-portraits clearly refer to the participant’s individual self, particularly when mirror-imaged self-portraits are used. This creates a high degree of self-relevance which is often lacking in more semantic tasks. Moreover, the Self-AAT does not require a choice between self-related versus other-related stimuli, as, for instance, the IAT does.^2^

Finally, it should be explored if and how the self-approach tendency can be experimentally modified, and whether an induced increase in self-approach has any positive effects on the trained individual. Recent studies in the area of cognitive bias modification indicate that a number of approach-avoidance tendencies can be trained in order to reduce emotional vulnerability (e.g., Rinck et al., 2013). Maybe individuals lacking self-approach could profit from a joystick task in which they are extensively trained to approach themselves. This will be a promising direction for further research.

## Data Availability

All data sets generated for this study are available at: https://osf.io/zxqk2/.

## Author Contributions

EB and MR created the approach-avoidance task. HG, EB, and MR designed Experiment 1, and HG conducted it under the supervision of MR and EB. LS designed, conducted, and analyzed Experiments 2 and 3 under the supervision of MR. All authors wrote parts of the original manuscript version, and the final version was written and revised by MR. All authors approved the final version of the manuscript for submission.

## Conflict of Interest Statement

The authors declare that the research was conducted in the absence of any commercial or financial relationships that could be construed as a potential conflict of interest.

## Footnotes

^1^Please note that this might not be true for whole-body pictures; here we might see photographs of ourselves as often as whole-body images in mirrors. However, these images were not the subject of our experiment.

^2^It should be noted, however, that even though the Self-AAT does not require a choice between self-related versus other-related stimuli, its resulting scores are still relative: The approach-avoidance tendency of interest must be compared to the tendency observed for control stimuli.
